# Narrative role of vitamin D receptor with osteoporosis and obesity in a sample of Egyptian females: a pilot study

**DOI:** 10.1186/s43141-021-00216-0

**Published:** 2021-08-05

**Authors:** Nayera E. Hassan, Sahar A. El-Masry, Waheba Ahmed Zarouk, Ghada Nour Eldeen, Rehab M. Mosaad, Mahmoud A. S. Afify, Manal M. Aly, Aya Khalil

**Affiliations:** 1 Biological Anthropology Department, Medical Research Division, National Research Centre, 33 El-Bohooth St., Dokki, Cairo, Giza 12622 Egypt; 2 Molecular Genetics and Enzymology Department, Human Genetics and Genome Division, National Research Centre, Cairo, Egypt

**Keywords:** Vitamin D receptor polymorphism, TaqI genotype, ApaI genotype, Osteoporosis, Obesity, Egyptian females

## Abstract

**Background:**

Vitamin D receptor (VDR) is known as one of the cellular regulators for several metabolic pathways indicating its pivotal role in the pathological pathway of numerous diseases. Considering the high frequency of osteoporosis and obesity among women, the current study aimed to explore the prospective assembly of the most frequent two *VDR* loci, single nucleotide polymorphism SNPs rs731236 (TaqI) and rs7975232 (ApaI) with a genetic predisposition to osteoporosis (skeletal) and obesity (chronic non-skeletal disorders), in Egyptian women. This was a cross-sectional study, including 97 Egyptian females (25–65 years), randomly chosen, from all employees and workers of the National Research Centre, Egypt. Anthropometric measurements (weight, height, BMI), dual-energy X-ray absorptiometry (DEXA), and molecular genetic analysis were done.

**Results:**

The variation of ApaI genotype between the normal and osteoporotic groups denotes a remarkable association of the homozygote ApaI genotype with osteoporosis risk. Among the normal weight group, bone mineral density (BMD) was significantly associated with TaqI VDR gene polymorphism as the presence of the heterozygote genotype was accompanied with higher BMD while the homozygote one was detected with lower BMD. Also, TaqI VDR gene polymorphism was significantly associated with BMI when participants were divided according to the presence of osteoporosis; increased BMI was expressed in the non-osteoporotic women group carrying the homozygote genotype of Taq I VDR gene while the presence of the heterozygote genotype (TaqI) in the osteoporotic group was associated with increased BMI.

**Conclusions:**

The heterozygote TaqI genotype is protective against the osteoporosis phenotype and accompanied with increased BMI among osteoporotic women, while the homozygote ApaI genotype has a significant association with osteoporosis risk.

## Background

Osteoporosis and obesity are worldwide health problems and greatly affecting public health, being often associated with high morbidity and mortality leading to reduced quality of life and increased economic cost [1]. Worldwide, there is a difference in gender as one-third of women above the age of 50 years were exposed to osteoporotic fractures in relation to one-fifth of men of the same age group [2]. The prevalence of obesity worldwide indicates a pandemic; the health and demographic survey enrolled by the Health Ministry in Egypt indicates that around 46.3% of females are obese [3]. The etiology of both diseases has been supposed to arise from dysregulation of bone marrow mesenchymal stromal cells which are considered as a common precursor cell for both osteoblasts and adipocytes [4].

Data originating from twin studies has reported that genetic factors represent up to 85% of diversity in bone mass [5] and the coincidence for fat mass among monozygotic (MZ) twins has been denoted to range from 70 to 90%, while in dizygotic (DZ) twins it is 35–45% [6].

Vitamin D metabolic functions occur through the binding of the active form of vitamin D receptor (VDR), 1, 25-dihydroxy-vitamin D (1, 25(OH)2D), to it. Then, the retinoic acid receptor (RXR) joins this complex, producing ultimately heterodimers that act on vitamin D response elements targeting gene promotor regions. A cascade of transcriptional regulations affecting target genes occurs. VDRs exist nearly in all human tissues including adipose tissue. They are mediating the function of vitamin D and so are essential for epigenome and expression of more than 1000 genes [7].

The human *VDR* gene is localized on chromosome 12q13.1 which spans ~ 75 kb genomic DNA and is presented with 11 exons [8]. Several VDR genetic polymorphisms are reported. The 3′ end region is part of the ligand-binding domain of the VDR. The reported polymorphisms such as that of ApaI and TaqI, which are located at the 3′ untranslated region (3′UTR) of the VDR gene, are the most prevalent and extensively studied genetic markers in relation to bone mineral density (BMD) variations in adult females as it influences the mRNA stability and VDR expression [9]. The structural protein variations secondary to ApaI and TaqI polymorphisms may lead to alternation of the binding specificity of vitamin D. Mutations in functional regions of the *VDR* gene affect the metabolism of minerals—especially calcium—and therefore bone density [10]. Variations in VDR genetic alleles have been demonstrated to be associated with metabolic syndrome (MS) and its components including anthropometric parameters related to obesity. Although the genetic background of obesity is complex, recently, it was evidenced that functional polymorphisms of certain genes might affect the whole interindividual susceptibility to obesity [11, 12].

On contrary, some studies have reported that there is no association between VDR gene polymorphisms and the risk for MS development [13–15]. This debate remains unclear and requires further large-scale studies.

evidence that; obesity and osteoporosis share some common genetic determinations and the fact that the VDR is widely distributed, is controlling genes related to bone metabolism, chronic diseases, and inflammation. Consequently, it is fundamental to verify, characterize, and correlate the occurrence of such genetic variations of the VDRs. This will propose a personalized clinical approach to prohibit or at least postpone the development of these chronic diseases and subsequent complications. From this point of view, this study was conducted to evaluate the genetic association of selected polymorphic variants within the *VDR* gene, particularly *ApaI* (rs7975232) and *TaqI* (rs731236), in obesity development and osteoporotic risk, among a sample of Egyptian females, a country where obesity is reaching endemic proportions.

## Methods

This was a cross-sectional study, which included 97 Egyptian women. Their ages ranged between 25 and 65 years with a mean age of 48.85 ± 9.88 years. They were recruited and randomly chosen, from all employees and workers, of all categories, of the “National Research Centre”, Egypt. All participated women were free from any chronic disease or under long-term medications. A written informed consent was obtained from all participants after being informed about the purpose of the study. This research paper was derived from a cross-sectional survey of a project funded by “National Research Centre”, Egypt, 2016–2019 entitled “Bone mass among Overweight and Obese Women: Mechanism and Intervention.” “National Research Centre”, with an approval obtained from the Ethics Committee of “National Research Centre” (registration number is 16/127).

For each participant woman, anthropometric measurements, dual-energy X-ray absorptiometry (DEXA) measurements, and molecular genetic analysis were done.

### Anthropometric measurements

Body weight and height were measured, following the recommendations of the “International Biological Program” [16]. Body weight (Wt) was determined to the nearest 0.01 kg using a Seca Scale Balance, with the woman wearing minimal clothes and with no shoes. Body height (Ht) was measured to the nearest 0.1 cm using a Holtain portable anthropometer. Body mass index (BMI) was calculated: [BMI: weight (in kilograms) divided by height (in meters squared)]. The participant women were classified according to their BMI into 2 groups: 31 women with normal BMI (<25 kg/m^2^) and 66 overweight/obese women (> 25 kg/m^2^).

### DEXA measurements

Both bone mineral density “BMD” (gm/cm^2^) and BMD *T* score at the neck of the femur were measured using dual-energy DEXA (DEXA Norland XR-46 version 3.9.6/2.3.1, USA). A full body DEXA scan, based on the woman’s age, weight, and height, was performed with the participant keeping the precise distance between her arms and legs according to the machine instruction manual. A well-qualified operator executed and evaluated all analyses using the same protocol for all assessments. According to the WHO diagnostic criteria [17] depending on BMD *T* score at any of the recommended sites (lumbar spine or femoral neck), the women were classified into 3 groups: women with healthy bone ( > −1), osteopenia (between −1 and > −2.5), and osteoporosis (<−2.5) [18]. After that, BMD *T* score −1 was taken to group the participating women to a non-osteoporotic or osteoporotic group.

### Molecular genetic analysis

#### Genomic DNA extraction

Genomic DNA was extracted using Qiagen QIAamp DNA Blood Mini Kit from whole blood samples according to the manufacturer’s protocol. The concentration of genomic DNA was determined by quantitative method, based on optical density measurement using NanoDrop UV/V (Thermo Scientific, UK). The purity of DNA was determined by calculating the ratio of absorbance at 260 nm to absorbance at 280 nm (*A*_260_/*A*_280_). Pure DNA should have an *A*_260_/*A*_280_ ratio of 1.7–1.9, respectively.

#### VDR gene polymorphism genotyping by PCR-restriction fragment length polymorphism (RFLP)

The ApaI and TaqI polymorphic sites of VDR were considered. The targeted SNP was amplified by conventional polymerase chain reaction (PCR) and followed by restriction digestion. The VDR genotype of each subject was identified according to the digestion pattern and alleles.

##### TaqI polymorphism

*PCR amplification*: PCR reaction was carried out in 25-μL reaction mixture containing 1.5 mM MgCl2, 0.2 mM dNTP, and 10 pmoles of each primer sequences F: 5′-CAG AGC ATG GAC AGG GAG CAA-3′ and R: 5′-CAC TTC GAG CAC AAG GGG CGT TAG C-3′ as described previously by Mohamed and El-Askary [19], 0.5 U of Taq DNA polymerase, and 200ng of genomic DNA. PCR conditioning was as follows: initial denaturation for 5 min at 95°C, 30 cycles of 30s at 94°C, annealing for 45s at 57°C, extension for 60s at 72°C, and a final extension for 5 min at 72 °C.

##### ApaI polymorphism

*PCR amplification*: PCR reaction was carried out in 25-μL reaction mixture containing 1.5 mM MgCl2, 0.2 mM dNTP, and 10 pmoles of each primer sequences F: 5′-CAA CCA AGA CTA CAA GTA CCG CGT CAG TGA-3′ and R: 5′-CAC TTC GAG CAC AAG GGG CGT TAG C-3′ as described previously by Mohamed and El-Askary [19], 0.5 U of Taq DNA polymerase, and 200ng of genomic DNA. PCR conditioning was as follows: initial denaturation for 5 min at 95°C, 30 cycles of 30s at 94°C, annealing for 45s at 55°C, extension for 60s at 72°C, and a final extension for 10 min at 72 °C.

*Post-PCR-RFLP*: The resulting DNA fragments were subjected to restriction digestion using respective enzymes ApaI and Taq-I (Promega, Madison, USA, 10 U/ml). The Eppendorf tubes for RFLP were prepared as follows: 10 μl of PCR product; 16.3 μl of sterile, deionized water; 0.2 μl of 100X BSA; and 2 μl of 10X RE Buffer, and mixed by pipetting. Finally, 10 units of each of the respective restriction enzymes were added. The tubes were incubated (2 h at 37°C for ApaI and Taq I polymorphisms) and heat inactivated for 15 min at 80°C. The genotypes were resolved on 2% (w/v) agarose gels.

**Fig. 1:**
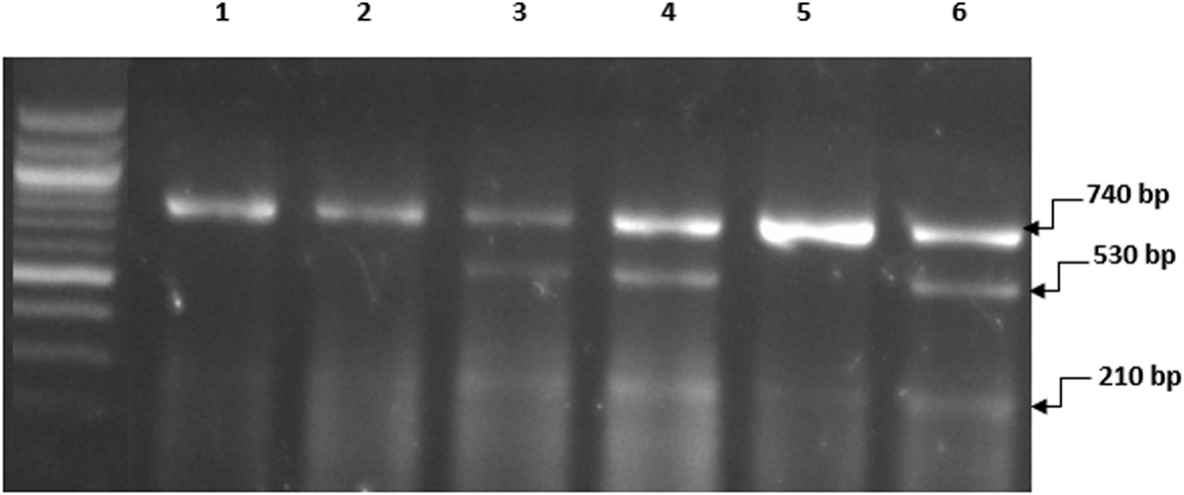
The genotypes of VDR gene polymorphism using ApaI restriction enzyme, 100-bp DNA marker (NEB). Lanes 1, 2, and 5: ApaI with homozygote genotype; uncleaved ApaI site fragment 740 bp. Lanes 3, 4, and 6: ApaI with heterozygote genotype; heterozygosis fragments 740, 530, and 210 bp

**Fig. 2:**
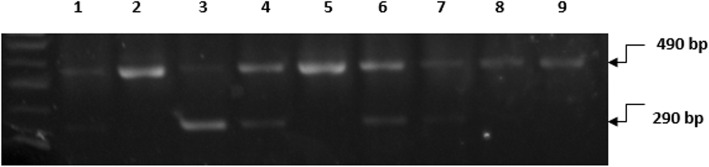
PCR-RFLP analysis of the VDR gene polymorphism, using TaqI restriction enzyme 100-bp DNA marker (NEB). Lanes 1, 3, 4, 6, and 7: heterozygous genotype; fragments; 495, 290, and 205 bp (undetected). Lanes 2, 5, 8, and 9: wild type homozygote

*3-Genotyping*: The genotypes were resolved on 2% (w/v) agarose gels. *ApaI polymorphism* genotyping for wild type homozygosis fragment (AA) at 740-bp mutant homozygosis fragments (aa) at 530 and 210 bp and heterozygosis produces fragment (Aa) of 740, 530, and 210 bp. In the presence of A-allele, there was no restriction enzyme cleavage site and a product of 740 bp was obtained. In subjects carrying “a-allele,” the cleavage products of 530 and 210 bp were detected. Alleles “A” and “a” were assigned based on the presence of a 740-bp (uncleaved) fragment and the 530-bp and 210-bp (cleaved) fragments, respectively (Fig. 1). *TaqI polymorphism* genotyping for wild type homozygosis produces fragment (TT) at 495 bp while heterozygosis produces fragments (Tt) at 495, 290, and 205 bp. The allele “T” was associated with the presence of a 495-bp fragment, while allele “C” was assigned in the presence of 290-bp and 205-bp fragments (Fig. 2).

### Statistical analysis

Data were analyzed using the Statistical Package for Social Sciences (SPSS/Windows version 16, SPSS Inc., Chicago, IL, USA). The normality of data was tested using the Kolmogorov-Smirnov test. The data of DEXA, weight, and BMI were not normally distributed. So, non-parametric tests were used.

The 97 participant women were classified twice into 2 groups: first according to their BMI (31 normal weight and 66 overweight/obese) and second according to their BMD *T* score (23 osteoporotic and 74 non-osteoporotic). The parametric data were expressed as mean ± SD, where the qualitative ones were expressed as number and percentage (%). The various parametric variables of the different groups were analyzed and compared using the Mann-Whitney test for independent groups, while the frequency distribution of the vitamin D receptors among different groups (non-parametric data) were compared using the chi-square test. *P* < 0.05 was regarded as statistically significant for all tests.

## Results

Frequency distribution of VDR gene polymorphisms (TaqI and ApaI) among non-osteoporotic and osteoporotic groups (Table 1) revealed that ApaI polymorphism was significantly associated with BMD (*P* = 0.012). The homozygote ApaI genotype was the most abundant among osteoporotic women (95.7%), while the heterozygote one was more frequent among non-osteoporotic women (35.1%). Meanwhile, the frequency distribution of VDR (ApaI and TaqI) genes among overweight/obese cases and the normal weight group (Table 2) showed insignificant differences in the distribution of VDR polymorphisms.

**Table 1:** Frequency distribution of TaqI and ApaI genotypes among non-osteoporotic and osteoporotic groups

Variables		Non-osteoporotic (***N*** = 74)		Osteoporotic (***N*** = 23)		Chi-square
		***N***	%	***N***	%	
**Vit. D receptor**						
**Taq1**	Homo	42	56.8	18	78.3	0.064
	Hetero	32	43.2	5	21.7	
**Apa1**	Homo	47	63.5	22	95.7	0.012*
	Hetero	26	35.1	1	4.3	
	Mutant	1	1.4	0	0	

N.B. *P < 0.05 = significant differences

**Table 2:** Frequency distribution of TaqI and ApaI genotypes among overweight/obese and normal weight groups

Variables		Overweight/obese (***N*** = 66)		Normal weight (***N*** = 31)		Chi-square
		***N***	%	***N***	%	
**Vit. D receptor**						
**Taq1**	Homo	43	65.2	17	54.8	0.330
	Hetero	23	34.8	14	45.2	
**Apa1**	Homo	44	66.7	25	80.6	0.329
	Hetero	21	31.8	6	19.4	
	Mutant	1	1.5	0	0	

Comparisons of the means ± SD of BMI and BMD at the femur neck between genotypes of Taq I and ApaI VDR gene polymorphisms among different groups are presented in Tables 3 and 4. Among the normal weight women (Table 3), the Taq I genotype of VDR had a significant effect on BMD value and osteoporotic risk, as the heterozygote genotype of TaqI VDR gene polymorphism had higher BMD than the homozygote one in the same group (*P* = 0.000).

**Table 3:** Comparisons of means ± SD of BMI and BMD between different genotypes of TaqI and ApaI VDR among overweight/obese and normal weight women

Variables	Taq1					Apa1				
	Overweight/obese (*N* = 66)									
	HOMO (*N* = 43)		Hetero (*N* = 23)		*P*	HOMO (*N* = 44)		Hetero (*N* = 22)		*P*
	Mean	±SD	Mean	±SD		Mean	±SD	Mean	±SD	
BMI	34.44	5.46	36.56	9.13	0.652	35.19	6.96	35.56	7.05	0.888
BMD *T* score at the femur neck	−1.40	1.16	−1.41	1.45	0.928	−1.38	1.36	−1.41	1.03	0.968
	Normal weight women (*N* = 31)									
	HOMO (*N* = 17)		Hetero (*N* = 14)		*P*	HOMO (*N* = 25)		Hetero (*N* = 6)		*P*
	Mean	+SD	Mean	+SD		Mean	±SD	Mean	±SD	
BMI	22.46	1.74	22.68	1.99	0.597	22.29	1.95	23.66	0.15	0.105
BMD *T* score at the femur neck	−2.90	0.47	−1.96	0.25	0.000**	−2.53	0.66	−2.23	0.17	0.314

N.B. *BMI* body mass index, *BMD* bone mineral density; *P < 0.01 = highly significant differences

**Table 4:** Comparisons of means ± SD of BMI and BMD between different genotypes of TaqI and ApaI VDR in non-osteoporotic and osteoporotic women

Variables	Taq1					Apa1				
	Non-osteoporotic women (*N* = 74)									
	HOMO (*N* = 42)		Hetero (*N* = 32)		*P*	HOMO (*N* = 47)		Hetero (*N* = 26)		*P*
	Mean	±SD	Mean	±SD		Mean	±SD	Mean	±SD	
BMI	33.47	6.15	30.65	8.88	0.032*	32.10	7.34	32.72	8.07	0.954
BMD *T* score at the femur neck	−1.34	1.12	−1.38	1.05	0.845	−1.24	1.15	−1.56	0.96	0.168
	Osteoporotic women (*N* = 23)									
	HOMO (*N* = 18)		Hetero (*N* = 5)		*P*	HOMO (*N* = 22)		Hetero (*N* =1 )		
	Mean	±SD	Mean	±SD		Mean	±SD	Mean	±SD	
BMI	25.39	6.31	35.50	15.91	0.046*	−3.011	0.410	−2.63		
BMD *T* score at the femur neck	−2.95	0.42	−3.16	0.34	0.446	27.12	9.70	37.97		

N.B. *BMI* body mass index, *BMD* bone mineral density; *P < 0.05 = significant differences

When groups were divided according to the presence or absence of osteoporosis (osteoporosis risk) (Table 4), BMI values differ by VDR gene polymorphisms in the case of TaqI and this difference was statistically significant (*P* = 0.032). BMI was increased among women carrying the homozygote genotype of Taq I VDR gene in the non-osteoporotic group, while increased BMI was associated with the heterozygote Taq I genotype in the osteoporotic group.

## Discussion

The abundant distribution of VDRs in skeletal and non-skeletal tissues and its existence in several cellular cascades indicates its crucial role in the pathophysiology of many diseases [20, 21]. Numerous studies investigated the association between *VDR* variants (*ApaI* and *TaqI*) and bone disorders and disparate results were detected. The current study found a significant association between ApaI VDR genotypes and osteoporosis in Egyptian women. This finding was supported and pooled in a large meta-analysis performed by Zhang et al. [22] who reported that there were significant correlations between VDR *Apa*I and postmenopausal osteoporosis susceptibility in the Caucasian populations and indicating that postmenopausal females having mutant allele of VDR ApaI might have less susceptibility to osteoporosis compared to those with wild genotype. Also, our results identified higher BMD among the normal weight women group carrying the heterozygote genotype of the VDR TaqI polymorphism than those with the homozygote one and this difference is statistically significant. In agreement with our results, in Belarusian osteoporotic postmenopausal women, both *ApaI* and *TaqI* genetic variations were found to be introducing factors of osteoporosis [23].

On contrary, a meta-analysis study by Shen et al. [24], of total 6500 osteoporotic women, showed that no association was found among the ApaI and TaqI polymorphisms and the prevalence of bone fracture. Also, Wang et al. [25] and Zhang et al. [22] conducted meta-analyses of 18 studies of VDR *TaqI* polymorphism and reported no significant relationship between *Taq*I polymorphism and osteoporosis incidence, while in Yadav et al. [26] meta-analysis, a total of 65 (14929 samples), 31 (7697 samples), 18 (3617 samples), and 26 (5353 samples) were studied. The authors found that the recessive model of *Taq*I polymorphism is associated with osteoporosis in the Caucasian population. The other polymorphism *Apa*I has no significant effect in low bone density. The prevalence of various VDR gene natural variants varies in numerous ethnic/regional populations. Due to this, the influence of these variations might vary from ethnic group to another. In addition, they provided that various gene to gene interactions and the epigenetic role of the environment should also be extensively studied in the future, as it could explain the genetics of osteoporosis [26]. Sakamoto et al. [27] study on 499 Japanese women showed that the VDR *Taq*I genotypes are significantly associated with bone mass in young Japanese women. Among them, the VDR *ApaI* heterozygote genotype is associated with increased bone mass concomitant with higher calcium intake.

In the current study, a significant association between TaqI *VDR* SNPs and obesity phenotype was found when studied groups were subdivided according to the presence of osteoporosis. Chen et al. [28] performed meta-analysis studies, including 1188 obese patients and 1657 healthy controls, to study the relationship between VDR polymorphisms and the incidence of obesity based on several case-control studies. Among them, the presence of the VDR ApaI natural variant with obesity susceptibility was investigated in 3 studies [11, 29, 30] and the TaqI polymorphism by Yiannis et al. [31], Fan et al. [29], Al Hazmi et al. [11], and Bienertová-Vašků et al. [30]. This meta-study reported that the T allele of TaqI could have a preservative role it could not find in the relationship between ApaI polymorphism and the incidence of obesity. Ruiz-Ojeda and his colleagues [32] hypothesized that allelic variations in the *VDR* gene might be potential participators in obesity pathophysiology through alternating adipocyte function and increasing adipocyte inflammation association. Some other studies denoted no association between VDR genetic variations and the susceptibility for MS development and its components including different elements of anthropometry related to obesity [13, 14]. In parallel, Karonova et al. [33] study found no significant relationship between VDR SNPs, rs7975232 (*ApaI*) and rs731236 (*TaqI*), and anthropometric of MS risk. Nam et al. [34] study on 506 Korean patients supports the association of VDR genetic variants and obesity risk.

In summary, the current study investigated the distribution of the VDR gene TaqI and ApaI variants’ effect on BMI and BMD among osteoporotic and non-osteoporotic women. A statistically significant difference was detected when the polymorphism genotype frequency was analyzed with regard to Taq I polymorphism only. BMI was significantly higher in non-osteoporotic women with the homozygote genotype than those with the heterozygote one, while in contrast the presence of this latter genotype (heterozygote TaqI) was accompanied with increased BMI in osteoporotic women. These observations are consistent with the view that phenotypes of various diseases may be the yields of interlinkage between genotypes and several environmental factors which may mask the genetic effects.

## Conclusions

The heterozygote TaqI genotype seems to be protective against the osteoporosis phenotype and it was accompanied with increased BMI among osteoporotic women, while the homozygote ApaI genotype has a significant association with osteoporosis risk. These polymorphisms may be considered useful markers for the screening of osteoporosis and obesity in certain ethnicities and may be potential targets for genetic therapy.

### Limitations

One of the main limitations in our work is the lack of control of confounding factors such as smoking. Second is the small sample size enrolled in the current study of Egyptian females; so, future studies in larger scale should focus on multiple haplotypes, to clarify the possible overall impacts of common VDR polymorphisms.

## Data Availability

The datasets used and/or analyzed during the current study are available from the corresponding author on reasonable request, after taking the permission of our institute “National Research Centre.”

## Abbreviations

1, 25(OH) 2D: 1, 25-Dihydroxy-vitamin D
BMD: Bone mineral density
BMI: Body mass index
DEXA: Dual-energy X-ray absorptiometry
DZ twins: Dizygotic twins
Ht: Body height
MS: Metabolic syndrome
MZ twins: Monozygotic twins
PCR: Polymerase chain reaction
SD: Standard deviation
SNPs: Single nucleotide polymorphisms
SPSS: Statistical Package for Social Sciences
RFLP: Restriction fragment length polymorphism
RXR: Retinoic acid receptor
Vit. D: Vitamin D
VDR: Vitamin D receptor
3′UTR: 3′ Untranslated region
WC: Waist circumference
Wt: Body weight
WHO: World Health Organization
